# Supplementary material for: What makes the Tc of monolayer FeSe on SrTiO3 so high: a sign-problem-free quantum Monte Carlo study
Source: Sci Bull (Beijing). 2016 Apr 30;61:925–30. doi: 10.1007/s11434-016-1087-x (PMC4914519; doi:10.1007/s11434-016-1087-x)
Supplement: Supplementary file 1 — Supplementary material 1 (pdf 97 KB) [file 11434_2016_1087_MOESM1_ESM.pdf]

Electronic Supplementary Materials for What makes the T_c of monolayer FeSe on $SrTiO_3$ so high: a sign-problem-free quantum Monte Carlo study

Zi-Xiang Li[1], Fa Wang[2,3], Hong Yao[1,3,*] & Dung-Hai Lee[4,5,*]
[1] *Institute for Advanced Study, Tsinghua University, Beijing 100084, China.*
[2] *International Center for Quantum Materials, School of Physics, Peking University, Beijing 100871, China.*
[3] *Collaborative Innovation Center of Quantum Matter, Beijing 100871, China.*
[4] *Department of Physics, University of California, Berkeley, CA 94720, USA.*
[5] *Materials Sciences Division, Lawrence Berkeley National Laboratory, Berkeley, CA 94720, USA.*

I. The effective action for the J_1 and J_2 types of spin fluctuations

The effective action, based on a two band model describing band structure of single-layer $(\mathrm{FeSe})_1$/STO, is given by $S = S_\mathrm{F} + S_\mathrm{s}$ where $S_\mathrm{s} = S_\mathrm{B} + S_\mathrm{FB}$ and

$$S_\mathrm{F} = \int_0^\beta \mathrm{d}\tau \sum_{jk,\alpha=x,y} \psi^\dagger_{j\alpha}\left[(\partial_\tau - \mu)\delta_{jk} - t_{jk,\alpha}\right]\psi_{k\alpha}, \tag{S1}$$

$$S_\mathrm{B} = \int_0^\beta \mathrm{d}\tau \Big\{ \frac{1}{2}\sum_j \frac{1}{c_\mathrm{s}^2}|\partial_\tau \vec{\varphi}_{\mathrm{s},j}|^2 + \frac{1}{2}\sum_{\langle jk\rangle}|\vec{\varphi}_{\mathrm{s},j} - \vec{\varphi}_{s,k}|^2 + \sum_j\left[\frac{r_\mathrm{s}}{2}|\vec{\varphi}_{\mathrm{s},j}|^2 + \frac{u_\mathrm{s}}{4}\left(|\vec{\varphi}_{\mathrm{s},j}|^2\right)^2\right]\Big\}, \tag{S2}$$

where

$$S_\mathrm{FB} = \lambda_\mathrm{s}\int_0^\beta \mathrm{d}\tau \sum_j (-1)^j\left[\psi^\dagger_{jx}(\vec{\sigma}\cdot\vec{\varphi}_{\mathrm{s},j})\psi_{jy} + h.c.\right], \tag{S3}$$

for J_1-type of spin fluctuations. If the spin fluctuation is J_2 type

$$S_\mathrm{FB} = i\,\lambda_\mathrm{s}\int_0^\beta \mathrm{d}\tau \sum_j (-1)^j\left[\psi^\dagger_{jx}(\vec{\sigma}\cdot\vec{\varphi}_{\mathrm{s},j})\psi_{jy} + h.c.\right]. \tag{S4}$$

In the above equations, j,k labels the sites of a square lattice, $\alpha = x,y$ labels the two orbitals (which transform into each other under the 90 ° rotation) from which the red and blue Fermi surfaces in Fig. 1a of the main text are derived from, τ denotes the imaginary time and β is the inverse temperature. In Eq. (S2), $\vec{\varphi}_\mathrm{s}$ is the AFM collective mode and the operator $\psi_{i\alpha}$ is a spinor operator which annihilates an electron in orbital α and on site i. The three $\vec{\sigma}$ are the spin Pauli matrices. It is important to note that in Eq. (S4) the fermion-boson coupling has an extra factor i.

The parameters in this effective action include r_s which tunes the system across the AFM phase transition, c_s is the spin-wave velocity and u_s is the self-interactions of $\vec{\varphi}_\mathrm{s}$. λ_s is the "Yukawa" coupling between electrons and AFM order parameter. In our computation, we fix $c_\mathrm{s} = u_\mathrm{s} = \lambda_\mathrm{s} = 1.0$ and vary the value of r_s to control the severity of AFM fluctuation. In the fermion action the hopping integral t_{ij} is chosen to be among nearest neighbor sites and equal to $t_\parallel = 1.0$ for $x(y)$-orbital along $x(y)$ direction and $t_\perp = -0.5$ for $y(x)$ orbital along $x(y)$ direction. We fix the occupancy in our computation to be 0.1 such that the Fermi surface is shown in Fig. 1a of the main text.

II. The effective action for antiferro-orbital fluctuations

The AFO effective action is given by $S = S_\mathrm{F} + S_\mathrm{o}$ where S_F is the same as in Eq.(S1), $S_\mathrm{o} = S_\mathrm{B} + S_\mathrm{FB}$ and

$$S_\mathrm{B} = \int_0^\beta \mathrm{d}\tau \Big\{ \frac{1}{2}\sum_i \frac{1}{c_\mathrm{o}^2}|\partial_\tau \varphi_{\mathrm{o},i}|^2 + \frac{1}{2}\sum_{\langle ij\rangle}|\varphi_{\mathrm{o},i} - \varphi_{\mathrm{o},j}|^2 + \sum_i\left[\frac{r_\mathrm{o}}{2}(\varphi_{\mathrm{o},i})^2 + \frac{u_\mathrm{o}}{4}\left(\varphi_{\mathrm{o},i}\right)^4\right]\Big\}, \tag{S5}$$

$$S_\mathrm{FB} = \lambda_\mathrm{o}\int_0^\beta \mathrm{d}\tau \sum_i (-1)^i\left[\varphi_{\mathrm{o},i}\psi^\dagger_{ix}\sigma_0\psi_{iy} + h.c.\right]. \tag{S6}$$

In Eq. (S6), φ_o is the AFO order parameter and σ_0 is the identity matrix in the spin space.

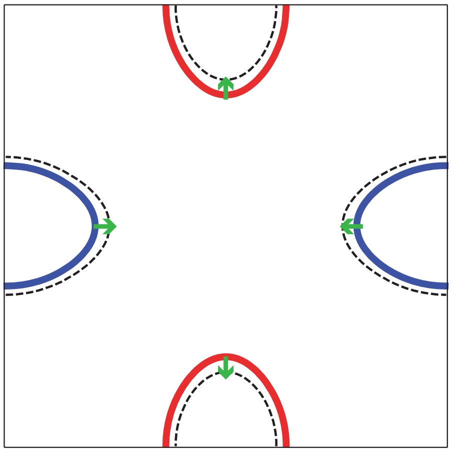

FIG. S1. Dashed lines represent the nematically distorted Fermi surfaces

The parameters in this effective action include the orbital wave velocity $c_{\rm o}$, and $r_{\rm o}$ which tunes the system across the AFO phase transition, and $u_{\rm o}$ is the self-interactions of the $\varphi_{\rm o}$ field. $\lambda_{\rm o}$ is the Yukawa coupling between electrons and AFO order parameter. In our computation, we fix $c_{\rm o} = u_{\rm o} = \lambda_{\rm o} = 1.0$ and vary the value of $r_{\rm o}$ to control the severity of AFO fluctuation.

III. The electron-phonon effective action

The electron-phonon effective action is given by $S = S_{\rm F} + S_{\rm ph}$ where $S_{\rm ph} = S_{\rm B} + S_{\rm FB}$ and

$$S_B = \int_0^\beta {\rm d}\tau \Big\{ \frac{1}{2}\sum_i \frac{1}{c_{\rm ph}^2}|\partial_\tau \varphi_{{\rm ph},i}|^2 + \frac{1}{2}\sum_{\langle ij\rangle} |\varphi_{{\rm ph},i} - \varphi_{{\rm ph},j}|^2 + \sum_i \Big[\frac{r_{\rm ph}}{2}|\varphi_{{\rm ph},i}|^2\Big]\Big\}, \tag{S7}$$

$$S_{\rm FB} = \lambda_{\rm ep}\int_0^\beta {\rm d}\tau \sum_i \varphi_{{\rm ph},i}\Big[\psi_{ix}^\dagger \sigma_0 \psi_{ix} + \psi_{iy}^\dagger \sigma_0 \psi_{iy}\Big]. \tag{S8}$$

Here $\varphi_{\rm ph}$ is the phonon field, $r_{\rm ph}$ is the frequency of the optical phonon at $\vec{q} = 0$ and $c_{\rm ph}$ is velocity of phonon. We fix parameters $r_{\rm ph} = 0.5, c_{\rm ph} = 1.0$ and vary the value of $\lambda_{\rm ep}$ to tune the strength of electron-phonon coupling.

IV. The effective action for nematic fluctuations

In Fig. S1, we show how does the nematic order parameter distort the Fermi surfaces. The nematic effective action is given by $S = S_{\rm F} + S_{\rm n}$ where $S_{\rm n} = S_{\rm B} + S_{\rm FB}$ and

$$S_{\rm B} = \int_0^\beta {\rm d}\tau \Big\{ \frac{1}{2}\sum_i \frac{1}{c_{\rm n}^2}|\partial_\tau \varphi_{{\rm n},i}|^2 + \frac{1}{2}\sum_{\langle ij\rangle} |\varphi_{{\rm n},i} - \varphi_{{\rm n},j}|^2 + \sum_i \Big[\frac{r_{\rm n}}{2}|\varphi_{{\rm n},i}|^2 + \frac{u_{\rm n}}{4}\varphi_{{\rm n},i}^4\Big]\Big\}, \tag{S9}$$

$$S_{\rm FB} = \lambda_{\rm n}\int_0^\beta {\rm d}\tau \sum_i \varphi_{{\rm n},i}\Big[\psi_{ix}^\dagger \sigma_0 \psi_{ix} - \psi_{iy}^\dagger \sigma_0 \psi_{iy}\Big]. \tag{S10}$$

In Eq. (S10), $\varphi_{\rm n}$ is the nematic order parameter. The parameters in this effective action include the velocity $c_{\rm n}$, and $r_{\rm n}$ which tunes the system across the nematic phase transition, and $u_{\rm n}$ is the self-interactions of the $\varphi_{\rm n}$ field. $\lambda_{\rm n}$ is the Yukawa coupling between electrons and nematic order parameter. In our computation, we fix $c_{\rm n} = u_{\rm n} = \lambda_{\rm n} = 1.0$ and vary the value of $r_{\rm n}$ to control the severity of nematic fluctuation.

V. The superconducting pair correlation function

To investigate superconductivity we calculate the equal time pair-pair correlation functions

$$P_{s/d}(\vec{r}_i) = \langle \Delta_{s/d}(\vec{r}_i)\Delta^\dagger_{s/d}(\vec{0})\rangle, \quad \text{(S11)}$$

where

$$\Delta_{s/d}(\vec{r}_i) = \psi^{\mathrm{T}}_{ix}(i\sigma_y)\psi_{ix} \pm \psi^{\mathrm{T}}_{iy}(i\sigma_y)\psi_{iy}, \quad \text{(S12)}$$

are the s (+ sign) and d (− sign) wave Cooper pair operators, respectively. To determine whether there is long-range order we put $\vec{r}_i$ to the maximum separation $\vec{x}_{\max}$ of the pair fields (for a system with linear dimension L the value of $\vec{x}_{\max}$ is $(L/2, L/2)$. Moreover, in order to minimize statistical errors, we average the correlation function over 25 sites around $\vec{x}_{\max}$. Thus the actual pair correlation we study is

$$\overline{P}_{d(s)}(\vec{x}_{\max}) = \frac{1}{25}\sum_{n,m=0,\pm1,\pm2} P_{d(s)}(\vec{x}_{\max} + n\hat{x} + m\hat{y}). \quad \text{(S13)}$$

VI. Effective actions that are amenable to sign-problem-free QMC simulation

The actions that are amenable to sign-problem-free QMC simulations are any mixture of $S_{\mathrm{F}} + c_1 S_s + c_2 S_{\mathrm{ph}} + c_3 S_{\mathrm{n}}$ and $S_{\mathrm{F}} + c_1 S_o + c_2 S_{\mathrm{ph}} + c_3 S_{\mathrm{n}}$ where $c_{1,2,3} = 0, 1$. Note, however, for S_{s} we can use either J_1 or J_2 type spin fluctuation actions but not both.

Aside from the square lattice spatial symmetries the action $S_{\mathrm{F}} + c_1 S_s + c_2 S_{\mathrm{ph}} + c_3 S_{\mathrm{n}}$ is invariant under the anti-unitary transformation $U = \tau_z(i\sigma_y)K$ and the action $S_{\mathrm{F}} + c_1 S_o + c_2 S_{\mathrm{ph}} + c_3 S_{\mathrm{n}}$ is invariant under $U' = i\sigma_y K$. Here τ_z is the third Pauli matrix acting in orbital (x, y) space and K denotes complex conjugation. It can be shown that because of these symmetries, the fermion determinant for arbitrary Bose fields configuration is positive hence the QMC simulation is free of minus-sign. This enables us to perform large-scale projector QMC simulation. The anti-unitary symmetry $U = \tau_z(i\sigma_y)K$ is the same as that in Ref. [1].

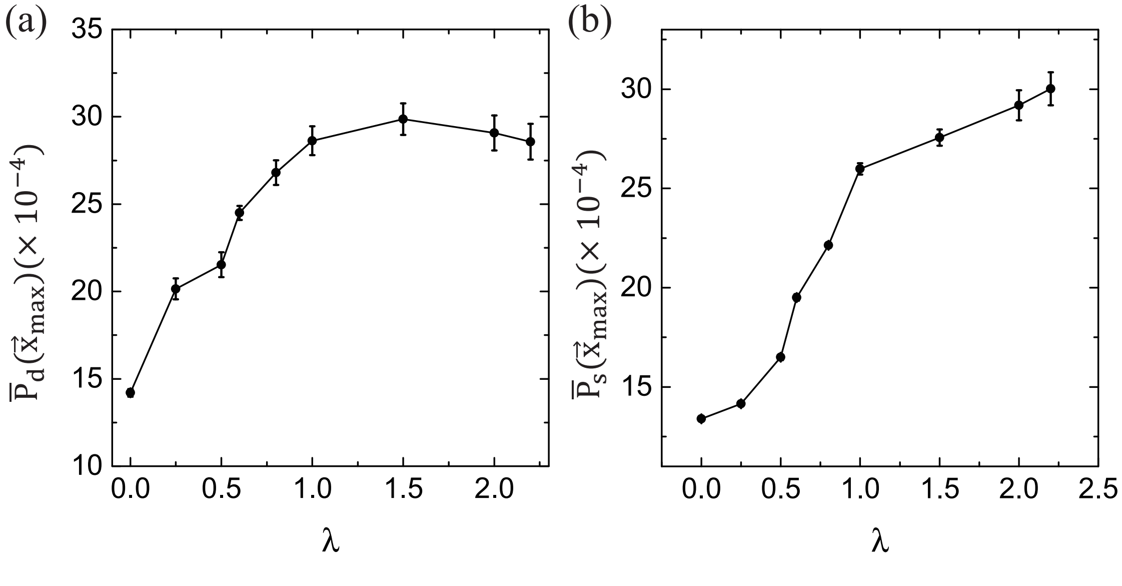

FIG. S2. Enhancement of pairing by electron-phonon coupling. **a** The J_1-type spin fluctuation triggered $\overline{P}_d(L/2, L/2)$ as a function of λ for $L = 14$. **b** The AFO fluctuation triggered $\overline{P}_s(L/2, L/2)$ as a function of λ for $L = 14$

VII. The enhancement of superconductivity triggered by the J_1 spin and AFO fluctuations as a function of the electorn-phonon coupling

In this section, we examine the enhancement of the SC order parameters triggered by the J_1 type spin and AFO fluctuations as a function of the dimensionless electron-phonon coupling strength λ.

In Fig. S2a and b, we plot the enhancement of spin fluctuation induced $\overline{P}_d(L/2, L/2)$ and orbital fluctuation induced $\overline{P}_s(L/2, L/2)$ as a function of λ for $L = 14$. Apparently the enhancement of superconductivity peaks at $\lambda \sim 1.5$ for the J_1-type spin fluctuation triggered d-wave pairing. For the AFO induced s-wave pairing the pair correlation increases monotonously with the amplitude of electron-phonon coupling up to the maximum value of λ we studied (=2.2).

* yaohong@tsinghua.edu.cn, dunghai@berkeley.edu.

[1] Berg E, Metlitski MA, Sachdev S (2012) Sign-problemCfree quantum Monte Carlo of the onset of antiferromagnetism in metals. Science 338:1606–1609
[2] Sorella S, Baroni S, Car R et al (1989) A novel technique for the simulation of interacting fermion systems. Europhys Lett 8:663
[3] White SR, Scalapino DJ, Sugar RL et al (1989) Numerical study of the two-dimensional Hubbard model. Phys Rev B 40:506
[4] Blankenbecler R, Scalapino DJ, Sugar R L (1981) Monte Carlo calculations of coupled boson-fermion systems. Phys Rev D 24:2278
[5] Assaad FF, Evertz HG (2008) World-line and determinantal quantum Monte Carlo methods for spins, phonons and electrons. In Computational many-prticle physics, Fehske H, Schneider R, Weiβe, A (Eds.). 277-356 (Lecture Notes in Physics, 2008)
